# Otophylloside B Protects Against Aβ Toxicity in *Caenorhabditis elegans* Models of Alzheimer’s Disease

**DOI:** 10.1007/s13659-017-0122-1

**Published:** 2017-02-13

**Authors:** Jie Yang, Xiao-Bing Huang, Qin-Li Wan, Ai-Jun Ding, Zhong-Lin Yang, Ming-Hua Qiu, Hua-Ying Sun, Shu-Hua Qi, Huai-Rong Luo

**Affiliations:** 1 State Key Laboratory of Phytochemistry and Plant Resources in West China, Yunnan Key Laboratory of Natural Medicinal Chemistry, Kunming Institute of Botany, Chinese Academy of Sciences, Kunming, 650201 Yunnan China; 2 University of Chinese Academy of Sciences, Beijing, 100049 China; 3 The Second Affiliated Hospital of Kunming Medical University, Kunming, 650101 Yunnan China; 4 Key Laboratory for Aging and Regenerative Medicine, Department of Pharmacology, School of Pharmacy, Southwest Medical University, Luzhou, 646000 Sichuan China; 5 Guangdong Key Laboratory of Marine Material Medical, South China Sea Institute of Oceanology, Chinese Academy of Sciences, Guangzhou, 510301 China

**Keywords:** Alzheimer’s disease, *Caenorhabditis elegans*, Otophylloside B, β-Amyloid peptide, HSF-1

## Abstract

**Abstract:**

Alzheimer’s disease (AD) is a major public health concern worldwide and the few drugs currently available only treat the symptoms. Hence, there is a strong need to find more effective anti-AD agents. *Cynanchum otophyllum* is a traditional Chinese medicine for treating epilepsy, and otophylloside B (Ot B), isolated from *C. otophyllum*, is the essential active component. Having previously identified anti-aging effects of Ot B, we evaluated Ot B for AD prevention in *C. elegans* models of AD and found that Ot B extended lifespan, increased heat stress-resistance, delayed body paralysis, and increased the chemotaxis response. Collectively, these results indicated that Ot B protects against Aβ toxicity. Further mechanistic studies revealed that Ot B decreased Aβ deposition by decreasing the expression of *Aβ* at the mRNA level. Genetic analyses showed that Ot B mediated its effects by increasing the activity of heat shock transcription factor (HSF) by upregulating the expression of *hsf*-*1* and its target genes, *hsp*-*12.6*, *hsp*-*16.2* and *hsp*-*70*. Ot B also increased the expression of *sod*-*3* by partially activating DAF-16, while SKN-1 was not essential in Ot B-mediated protection against Aβ toxicity.

**Graphical Abstract:**

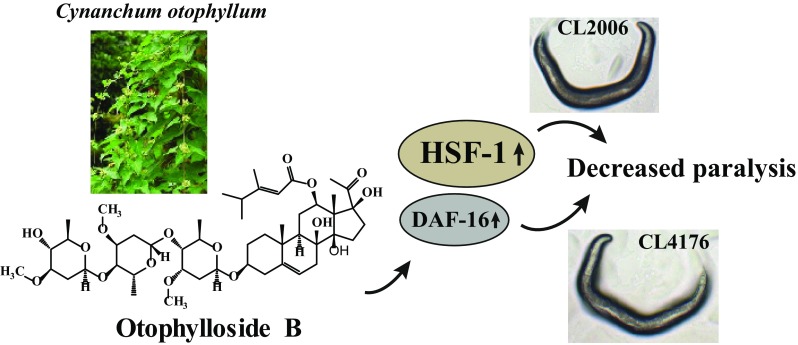

**Electronic supplementary material:**

The online version of this article (doi:10.1007/s13659-017-0122-1) contains supplementary material, which is available to authorized users.

## Introduction

Alzheimer’s disease (AD) is a neurodegenerative disorder that is strongly related to aging. As the number one cause of senile dementia, it is now becoming a major public health concern around the world [1]. Paradoxically, there are only a few drugs approved for the AD treatment, and these drugs only treat the symptoms. To date, there are still no disease-modifying drugs available, and there is a strong need to find more effective anti-AD drugs [2].

Although the etiology of AD remains unclear, a growing body of evidence implicates that the extracellular senile plaques that result from accumulation of β-amyloid (Aβ) and intracellular tau protein tangles are key histopathological hallmarks of Alzheimer’s disease (AD), and that the neurotoxicity of Aβ may play a central role in the pathogenesis of AD [3, 4].

Because of its short lifespan and amenability to genetic manipulation, *Caenorhabditis elegans* models that mimic human disease have been extensively used to study the disease mechanism and to screen potential drugs [5]. To study the neurotoxicity of Aβ, multiple transgenic *C. elegans* strains expressing the human Aβ_1–42_ peptides in either neurons or muscle cells have been constructed. For example, CL2006 has muscle-specific expression of Aβ, leading to a progressive paralysis that starts in adulthood. CL4176 expresses Aβ in muscle cells in temperature-sensitive manner [6], while CL2355 expresses Aβ in the neurons, which may more accurately represent the amyloid induced toxicity seen in AD [7].

Qingyangshen (*Cynanchum otophyllum*) is a traditional Chinese medicine, whose root is used for the treatment of epilepsy, rheumatic pain, kidney weakness and muscle injuries [8, 9]. Otophylloside B (Ot B) is a C-21 steroidal glycoside, the essential active ingredient of Qingyangshen. Ot B has been shown to inhibit the seizure-like locomotor activity of zebrafish [10] and extends the lifespan of wild type worms [11]. Since nutraceuticals with pro-longevity properties often have the potential to delay the onset of AD [12–16], we are wondering if Ot B could delay the Aβ-induced pathological behavior in *C. elegans*.

Here, we used several AD transgenic *C. elegans* models to evaluate the potential of Ot B for the prevention of AD and to determine its molecular mechanism of action. Our results showed that Ot B extended the lifespan, improved the heat resistance, dramatically improved Aβ-induced pathological behavior, for example, delayed the progression of body paralysis, and improved chemotaxis response. These results indicated that Ot B played a protective role against Aβ toxicity. Further results showed that Ot B reduced the accumulation of Aβ, probably by increasing the gene expression of several heat shock proteins (HSP).

## Results and Discussion

### Ot B Extends Lifespan and Increases Heat Stress Resistance in *C. elegans* with Muscle-Specific Expression of Aβ

**Fig. 1:**
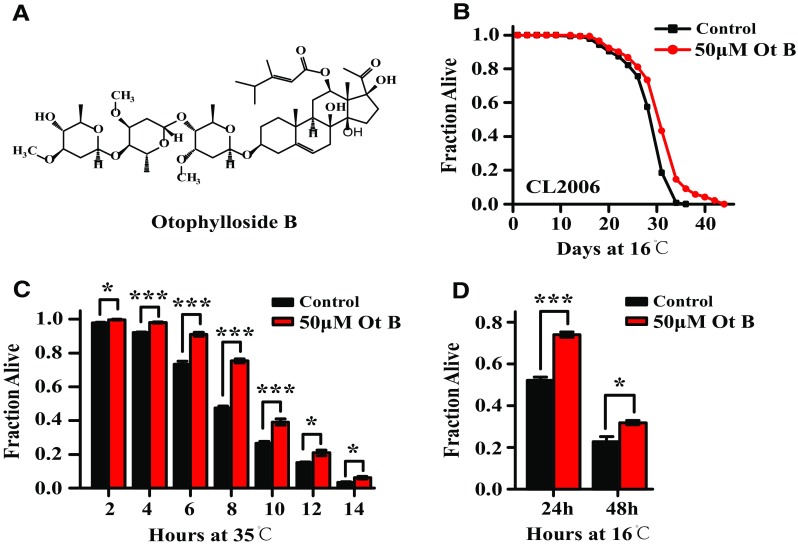
Otophylloside (Ot B) extended lifespan and improved thermo-tolerance in a *C. elegans* model of AD. **a** Chemical structure of Ot B. **b** Survival curves of CL2006 animals treated with vehicle or 50 μM of Ot B. **c** Survival percentage of CL2006 animals in heat resistance experiments. Heat resistance experiments were carried out at 35 °C and calculated by 2 h. **d** Survival percentage of CL2006 animals in heat resistance recovery experiment. The experiments were carried out at 35 °C for 7 h, then transferred to 16 °C and the number of dead worms was measured after 24 and 48 h. All the assays were carried out in triplicate, and at least three independent trials were performed. The columns showed the mean value of three independent experiments with *error bars* representing SEM. *** represents *p* < 0.001, * represents *p* < 0.05, calculated using two-tailed t test. Statistical details and repetition of this experiment are summarized in Table S1, S2 (Supplementary information)

Treatment of CL2006 worms having muscle-specific expression of Aβ with 50 μM of Ot B caused a significant increase in their lifespan compared with controls (*p* < 0.005; Fig. 1b, Supplementary Table 1). We also measured the effect of Ot B on heat stress resistance and heat recovery in CL2006 worms. Ot B treatment suppressed the lethality of heat stress in heat resistance experiments and heat resistance recovery experiments (*p* < 0.005; Fig. 1c, d, Supplementary Table 2). Together, these results indicated that Ot B slows aging and delays age-related degeneration in *C. elegans* with muscle-specific expression of Aβ.

### Ot B Delays the Progression of Body Paralysis, and Improves Chemotaxis Response

**Fig. 2:**
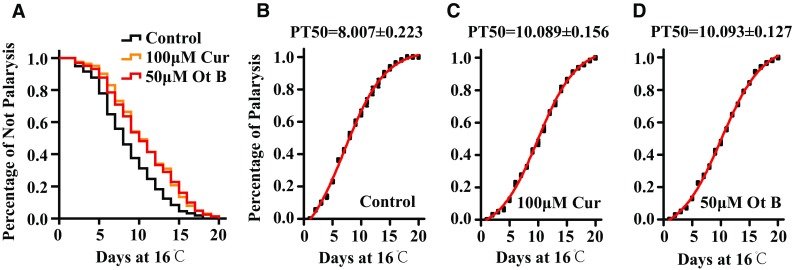
Ot B delayed progression of body paralysis in CL2006 worms. **a** Survival curves of control, Ot B treated and Cur treated animals. **b** Percentage of paralysis of control animals. **c** Percentage of paralysis of Cur treated animals. **d** Percentage of paralysis of Ot B treated animals. * Each bar* represents the mean of four independent experiments with* error bars *representing SEM. Each paralysis assay was conducted in triplicate and four trials were performed. Statistical details and repetition of this experiment are summarized in Table S3 (Supplementary information)

Paralysis is an apparent symptom of AD, and in *C. elegans* models of AD, it is a measurable phenotype that is considered as a result of Aβ toxicity [17]. Our paralysis assay with CL2006 showed that Ot B delayed paralysis by 21.4%, significantly increasing the PT_50_ from 8.0 to 10.1 days, which is comparable to 10.1 days in the curcumin-treated positive control group (*p* < 0.005; Fig. 2a, b, c, Supplementary Table 3).

**Fig. 3:**
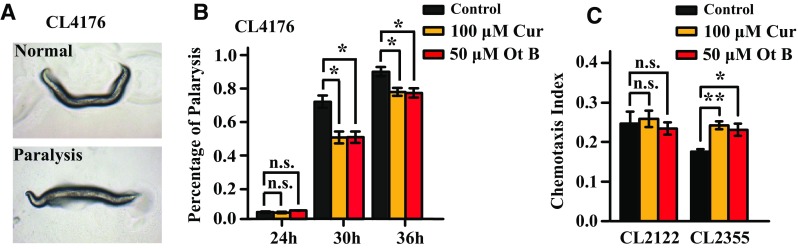
Ot B delayed progression of body paralysis in CL4176 and increased the chemotaxis response in CL2355 worms. **a** Photomicrograph showing CL4176 worms were paralyzed in larval animals. **b** Percentage of paralysis in CL4176 animals treated or non-treated with Ot B. 100 µM Curcumin was used as a positive control. Worms were grown at 16 °C for 48 h, and then transferred to 25°C. Paralysis experiments were carried out at 25 °C and calculated the paralysis of worms in 24, 30 and 36 h. **c** Chemotaxis index of CL2355 and its vector control, CL2122. Each *bar* represents the mean of three independent experiments with *error bars* representing SEM. ** represents *p* < 0.01,* represents *p* < 0.05, calculated using two-tailed t test. Statistical details and repetition of this experiment are summarized in Table S4, S5 (Supplementary information)

Our previous study showed that Ot B delayed aging and aging-related disorders [11], we were wondering if the delayed onset of paralysis was the result of an anti-aging effect, rather than a reduction in toxicity. To distinguish between these two hypotheses, we performed the paralysis assay in CL4176 worms, which express Aβ in muscle cells in a temperature-sensitive manner, leading to paralysis in larval animals (Fig. 3a) [18, 19]. At 30 h post temperature up-shift, Ot B decreased the paralysis from 73.3% to 51.1%, and 36 h later, 92.1% of the untreated worms became paralyzed, while only 79.4% of Ot B-treated and 78.7% of curcumin-treated positive control worms were paralyzed (*p* < 0.05; Fig. 3b, Supplementary Table 4). Together, these results indicated that Ot B delayed paralysis in young adults, and that this beneficial effect did not result from anti-aging.

The CL2355 strain expresses Aβ in the neurons, which may more accurately represent toxicity of Aβ seen in AD [20]. The chemotaxis response in *C. elegans* is mediated by interneurons to stimulate the motor neurons [21]. To determine if Ot B could protect against Aβ toxicity to neurons, we performed the chemotaxis response assay in CL2355 worms, using the CL2122 strain as a vector control. The response is reported as a chemotaxis index. The results for the vector control (CL2122) showed no difference between Ot B treated, curcumin positive controls, and untreated worms. In CL2355 worms, Ot B significantly improved the chemotaxis response (*p* < 0.05; Fig. 3c, Supplementary Table 5). These results indicated that Ot B protected against Aβ toxicity to neurological functions.

### Ot B Decreases Aβ Deposition in *C. elegans* Model of AD by Downregulating the Expression of *Aβ*

**Fig. 4:**
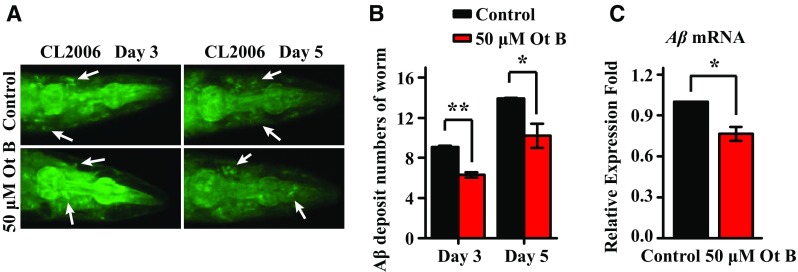
Ot B decreased Aβ deposition by depressing expression of *Aβ*. **a** Thioflavin S staining of CL2006. Ot B treated CL2006 worms were stained with thioflavin S on day 3 and day 5. White arrows indicate Aβ deposits. **b** Number of Aβ deposits in the worm head region. 50 μM Ot B significantly reduced the number of Aβ deposits in CL2006 both at day 3 and day 5. **c** The transcript level of *Aβ*, measured by qRT-PCR. The transcript level of Aβ was significantly downregulated by Ot B. The data was normalized to the expression of *cdc*-*42*.* Each bar *represents the mean value of three independent experiments with* error bars *representing SEM. ** represents *p* < 0.01,* represents *p* < 0.05, calculated using two-tailed t test. Statistical details and repetition of this experiment are summarized in Table S6, S7 (Supplementary information)

In CL2006 worms with muscle-specific Aβ expression, Aβ deposits are immunoreactive to anti-Aβ antibodies, and then these deposits bind to thioflavin S to produce deposits that can be viewed by confocal microscopy (Fig. 4a) [13]. To investigate if Ot B has a direct impact on the formation of Aβ, we conducted a thioflavin S staining experiment. The number of Aβ deposits was scored in the worm head region. The results showed that the mean number of Aβ deposits per nematode was significantly reduced in CL2006 worms treated with Ot B, compared with untreated worms at both day 3 and day 5 (*p* < 0.05; Fig. 4b, Supplementary Table 6). We next performed qRT-PCR to examine the effect of Ot B on the expression of *Aβ*. Ot B significantly reduced *Aβ* expression compared to untreated controls (*p* < 0.05; Fig. 4c, Supplementary Table 7). Collectively, these results showed the protection of Ot B against Aβ toxicity may result from the ability to decrease Aβ deposition by downregulating the expression of *Aβ* at the mRNA level.

### Ot B Alleviates Aβ Toxicity Mainly Through the HSF-1 Transcription Factor

Previous studies in *C. elegans* models of AD showed that the transcription factors DAF-16, SKN-1, and HSF-1 were involved in Aβ deposition [22, 23]. We performed qRT-PCR to test whether these regulators were involved in Ot B protection against Aβ toxicity.

**Fig. 5:**
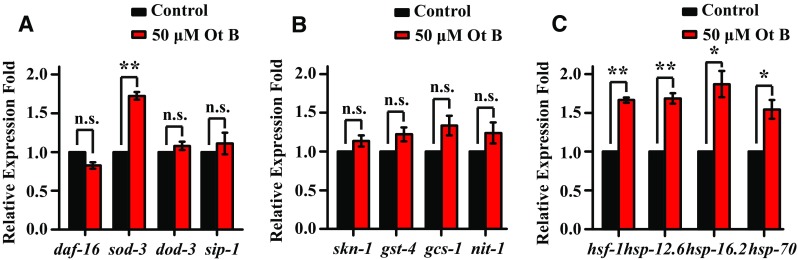
Gene expressions of transcription factors involved in Aβ deposition in controls and Ot B-treated *C. elegans*. **a** qRT-PCR measurement of mRNA transcripts for *daf*-*16* and its targeted genes *dod*-*3, sip*-*1, sod*-*3.* **b** qRT-PCR measurement of mRNA transcripts for *skn*-*1* and its targeted genes *gst*-*4, gcs*-*1* and *nit*-*1.* **c** qRT-PCR measurement of mRNA transcripts for *hsf*-*1* and its major target genes *hsp*-*12.6*, *hsp*-*16.2* and *hsp*-*70.* qRT-PCR was carried out using $2^{{ - }{{\Delta \Delta {\text{C}}_{\text{T}} }}}$ method and normalized to the expression of gene *cdc*-*42.* In each experiment, control and experimental worms were conducted in parallel and repeated in three independent trials. *Bars* represent the mean value of three independent experiments with* error bars *representing SEM. ** represents *p* < 0.01,* represents *p* < 0.05, calculated using two-tailed t test. Statistical details and repetition of this experiment are summarized in Table S7 (Supplementary information)

We found no difference in the expression of *daf*-*16* and its target genes, *dod*-*3* and *sip*-*1* between non-treated and treated worms, while the expression of another target gene, *sod*-*3* was significantly upregulated (Fig. 5a, Supplementary Table 7). We speculated that DAF-16 may play a partial role in Ot B -mediated protection against Aβ toxicity. Meanwhile, there was no difference observed in the expression of *skn*-*1* and its target genes, *gst*-*4, gcs*-*1* and *nit*-*1*. This may indicate that SKN-1 is not essential in Ot B -mediated protection against Aβ toxicity (Fig. 5b, Supplementary Table 7).

HSF-1 was reported to alleviate Aβ toxicity by disaggregating and degrading large Aβ aggregates into peptides or amino acids [24]. Our results showed that the treatment of Ot B significantly upregulated the expression of *hsf*-*1* and its targeted genes *hsp*-*12.6, hsp*-*16.2* and *hsp*-*70* (*p* < 0.05; Fig. 5c, Supplementary Table 7). Thus, heat shock protein (HSP) may be essential in Ot B-mediated protection against Aβ toxicity.

## Conclusion

Otophylloside B (Ot B), a C-21 steroidal glycoside, is an essential active ingredient of Qingyangshen (*C. otophyllum*). We recently demonstrated anti-aging effects of Ot B in *C. elegans*, and it has been reported that Ot B is neuroprotective in epilepsy [9, 11]. Our present study showed that Ot B extended lifespan in a *C. elegans* model of AD, increased heat stress-resistance, delayed the process of paralysis, and increased the chemotaxis response. Collectively, these results indicated that Ot B protected against Aβ toxicity. The Aβ deposition assay and gene expression experiment showed this may result from the ability of Ot B to decrease Aβ deposition by down-regulating the expression of *Aβ*. The molecular mechanism study revealed that Ot B up-regulated the expression of several heat shock proteins (HSP), including *hsf*-*1* and its target genes of *hsp*-*12.6*, *hsp*-*16.2* and *hsp*-*70.* It may also increase the expression of *sod*-*3* by partially activating the DAF-16, while SKN-1 was not essential in Ot B -mediated protection against Aβ toxicity. Taken together, these results indicate that Ot B has strong potential for development as a drug for AD prevention.

## General Experimental Procedures

### Chemicals and Strains

All strains were obtained from the Caenorhabditis Genetics Center (CGC) and maintained on NGM plates seeded with *Escherichia coli* OP50 at 16 °C. The following strains were used in this study: CL4176 *dvIs27[myo*-*3::Aβ*_*3*–*42*_ *let 3’UTR(pAF29); pRF4 (rol*-*6(su1006))]*, CL2006 *dvIs2[pCL12(unc*-*54::Aβ*_*1*–*42*_ *:)* + *pRF4]*, CL2122 *dvIs15[(pPD30.38) unc*-*54(vector)* + *(pCL26) mtl*-*2::GFP]*, and CL2355 *dvIs50[pCL45(snb*-*1::Aβ*_*1*–*42*_ *::3’ UTR(long)* + *mtl*-*2::GFP]I*.

Ot B was dissolved in DMSO for storage and diluted in PBS to a concentration of 50 µM while in use. Then the dilutions were overlaid onto the NGM plates. The final DMSO concentration was 0.1% after adding the drugs to the plates, and the negative control group had the same concentration of DMSO.

### Lifespan Assay

The lifespan assays were carried out in CL2006 at 16 °C. The strain was cultured for 2–3 generations before using for lifespan analysis. Lifespan assay were conducted as described previously [25]. In brief, for each assay, at least 40 synchronous L4 larvae or young adults were transferred to NGM plates containing inactivated OP50 (65 °C for 30 min), treated with 40 µM of FUdR to inhibit the growth of progeny and scored every other day. Animals were transferred to fresh plates with or without drugs every 2–4 days. All assays were carried out in triplicate, and at least three independent trials were performed. Statistics were calculated by using an SPSS package. The mean lifespan values were calculated by a log-rank (Kaplan–Meier) statistical test, with *p* < 0.05 accepted as statistically significant.

### Heat Resistance Assay

For heat resistance assays, synchronous strains of CL2006 at L4 stage or young adults were transferred to plates with or without Ot B and incubated at 35 °C. Dead animals were counted every 2 h. For heat resistance recovery assays, synchronous animals of CL2006 at L4 stage or young adults were transferred to plates with or without Ot B at 35 °C for 7 h, then transferred to 16 °C and dead animals were counted daily [26]. For each assay, at least 30 synchronous nematodes were studied, and three independent trials were performed. For statistical analysis, *p* values were calculated by a two-tailed *t* test, each consisting of control and experimental animals at the same time.

### Worm Paralysis Assay

For the paralysis assay of the CL4176 strain,late L3 larvae were grown at 16 °C for 48 h, then transferred to 25 °C to induce the expression of Aβ. Paralysis experiments were carried out at 25 °C. Calculation of the numbers of paralyzed worms was done at 24, 30 and 36 h after the transfer to 25 °C. For CL2006, paralysis experiments were carried out at 16 °C. Worms were checked every day until all worms were paralyzed. Worms were scored as paralyzed if they exhibited “halos” of cleared bacteria around their heads or moved their head only or did not move at all when they were gently touched by platinum worm pick [19]. Curcumin (100 µM) was used as a positive control. For each assay, at least 30 synchronous L4 larvae or young adult nematodes were studied. All paralysis plots were done in triplicate and three independent trials were performed. Statistics were calculated by using an SPSS package. The mean paralysis was calculated by a log-rank (Kaplan–Meier) statistical test, with *p* < 0.05 accepted as statistically significant.

### Chemotaxis Assay

CL2355 and its vector control CL2122 were used in chemotaxis assays. Synchronized L1 larvae were treated with Ot B or the vehicle. Chemotaxis experiments were carried out at 23 °C as described previously [27]. Briefly, worms were placed to the center of the plate, and 1 μL 0.1% benzaldehyde in 100% ethanol a with 1 μL of 1 M sodium azide was placed on one side of the plate, and 1 μL 100% ethanol along with 1 μL of 1 M sodium azide was placed on the opposite side. Curcumin (100 µM) was used as a positive control. The chemotaxis index was defined as follows: (number of worms at the attractant location – number of worms at the control location)/total number of worms on the plate). A two-tailed *t* test was used to calculate *p* values.

### Aβ Deposition Assay

For the Aβ deposition assay, CL2006 transgenic nematodes were fixed in 4% paraformaldehyde/PBS, pH 7.4, for 24 h at 4 °C, and then permeabilized in 5% fresh β-mercaptoethanol, 1% Triton X-100, 125 mM Tris pH 7.4, in a 37 °C incubator for 24 h. The worms were stained with 0.125% thioflavin S (Sigma) in 50% ethanol for 2 min, destained for 2 min in 50% ethanol, washed with PBS and mounted on slides for microscopy. Fluorescence images were acquired using a 40× objective of a fluorescence microscope. The Thioflavin S-reactive deposits anterior of the pharyngeal bulb in individual animals were scored [22]. A two-tailed test was used to calculate *p* values.

### Gene Expression Analysis by q-Real-Time PCR

Synchronized CL2006 L1 larvae were transferred to NGM plates cultured with or without Ot B and incubated at 16 °C. Worms in the young adult stage were collected with M9 buffer, then total RNA was extracted using RNAiso Plus (Takara) and converted to cDNA with a High Capacity cDNA Reverse Transcription Kit (Applied Biosystems). The cDNA of candidate genes were amplified and quantified in a Power SYBR Green PCR Master Mix (Applied Biosystems) on an ABI 7500 DNA analyzer (Applied Biosystems). Relative fold-changes for transcripts were calculated using $2^{{ - }{{\Delta \Delta {\text{C}}_{\text{T}} }}}$ method, and normalized to *cdc*-*42*. The experiments were conducted in triplicate. The data were analyzed using a two-tailed *t* test, and a *p* value <0.05 was accepted as statistically significant.

## Electronic supplementary material

Below is the link to the electronic supplementary material.

Supplementary material 1 (DOCX 49 kb)
