# Supplementary material for: Otophylloside B Protects Against Aβ Toxicity in Caenorhabditis elegans Models of Alzheimer’s Disease
Source: Nat Prod Bioprospect. 2017 Feb 13;7(2):207–14. doi: 10.1007/s13659-017-0122-1 (PMC5397390; doi:10.1007/s13659-017-0122-1)
Supplement: Supplementary file 1 — Supplementary material 1 (DOCX 49 kb) [file 13659_2017_122_MOESM1_ESM.docx]

**Supplementary information**

**Contents:**

**Table S1. The effectof Ot B on lifespan**

**Table S2. The effects of Ot B on heat resistance of CL2006**

**Table S3. The effects of Ot B on paralysis of CL2006**

**Table S4. The effects of Ot B on paralysis of CL4176**

**Table S5.** **The effects of Ot B on chemotaxis index of CL2122 and CL2355**

**Table S6. The effects of Ot B on Aβ deposition**

**Table S7. The effects of Ot B on mRNA expression**

**Table S8. Primer sequences of genes used in experiment**

**Table S1. The effects of Ot B on lifespan of CL2006**

| **Figure** | **Strains** | **Treatments** | **Mean Lifespan±SEM(days)** | **P value VS Control** | **% Change in Mean Lifespan** | **N** |
| --- | --- | --- | --- | --- | --- | --- |
|  | **CL2006** | | | | | |
| 1(B) | EXP.1 | 16℃/Control | 29.111±0.408 |  |  | 135 |
|  | EXP.1 | 16℃/50 µM Ot B | 31.126±0.461 | <0.001 | 6.922 | 143 |
|  | EXP.2 | 16℃/Control | 29.179±0.411 |  |  | 145 |
|  | EXP.2 | 16℃/50 µM Ot B | 30.521±0.448 | 0.008 | 4.600 | 117 |
|  | EXP.3 | 16℃/Control | 29.573±0.403 |  |  | 143 |
|  | EXP.3 | 16℃/50 µM Ot B | 30.821±0.294 | 0.013 | 4.220 | 134 |

The mean lifespan values were calculated by a log-rank (Kaplan-Meier) statistical test. *p* Values were calculated for individual experiments, each consisting of control and experimental animals as the same time. N: total number of worms in each individual experiment. All statistical were calculated by using SPSS package.

**Table S2. The effects of Ot B on heat resistance of CL2006**

| **Figure** | **Strains** | **Treatments** | **Mean Lifespan±SEM(hours)** | **P value VS Control** | **% Change in Mean Lifespan** | **N** |
| --- | --- | --- | --- | --- | --- | --- |
|  | **CL2006** | | | | | |
| 1(C) | EXP.1 | 35℃/Control | 9.158±0.362 |  |  | 76 |
|  | EXP.1 | 35℃/50 µM Ot B | 10.819±0.305 | 0.002 | 18.142 | 83 |
|  | EXP.2 | 35℃/Control | 9.130±0.403 |  |  | 69 |
|  | EXP.2 | 35℃/50 µM Ot B | 10.528±0.347 | 0.028 | 15.304 | 72 |
|  | EXP.3 | 35℃/Control | 9.111±0.383 |  |  | 72 |
|  | EXP.3 | 35℃/50 µM Ot B | 10.693±0.316 | 0.008 | 17.366 | 75 |
|  | EXP.4 | 35℃/Control | 9.096±0.368 |  |  | 73 |
|  | EXP.4 | 35℃/50 µM Ot B | 10.400±0.292 | 0.036 | 16.287 | 75 |

| **Figure** | **Strain** | **Treatment** | **24h** | **48h** | **N** |
| --- | --- | --- | --- | --- | --- |
|  | **CL2006** | | | | |
| 1(D) | EXP.1 | 16℃/Control | 0.486 | 0.157 | 70 |
|  | EXP.1 | 16℃/50 µM Ot B | 0.736 | 0.306 | 72 |
|  | EXP.2 | 16℃/Control | 0.521 | 0.247 | 73 |
|  | EXP.2 | 16℃/50 µM Ot B | 0.764 | 0.333 | 77 |
|  | EXP.3 | 16℃/Control | 0.565 | 0.246 | 69 |
|  | EXP.3 | 16℃/50 µM Ot B | 0.704 | 0.296 | 71 |
|  | EXP.4 | 16℃/Control | 0.514 | 0.264 | 72 |
|  | EXP.4 | 16℃/50 µM Ot B | 0.757 | 0.338 | 74 |
| **Mean Lifespan** | | **Control** | 0.521 | 0.228 |  |
| **SEM** | |  | 0.016 | 0.024 |  |
| **Mean Lifespan** | | **50 µM Ot B** | 0.740 | 0.318 |  |
| **SEM** | |  | 0.013 | 0.010 |  |
| **P value VS Control** | |  | 0.000 | 0.013 |  |

Heat resistance experiments were carried out at 35℃ and calculated by 2h. The mean lifespan and *p* Values were calculated as described for Table S1. N: total number of worms in each individual experiment.

Heat resistance recovery experiments were carried out at 35℃ for 7h, then transferred to 16℃ and calculated the dead after 24h and 48h. *p* Values were calculated by two-tailed t-test, each consisting of control and experimental animals as the same time. N: total number of worms in each individual experiment.

**Table S3. The effects of Ot B on paralysis of CL2006**

| **Figure** | **Strains** | **Treatments** | **Mean Lifespan±SEM(days)** | **P value VS Control** | **% Change in Mean Lifespan** | **PT50**  **(days)** | **N** |
| --- | --- | --- | --- | --- | --- | --- | --- |
|  | **CL2006** | | | | | | |
| 2(A) | EXP.1 | 16℃/Control | 8.572±0.314 |  |  | 7.807 | 180 |
| 2(B) | EXP.1 | 16℃/100µM Cuc | 10.339±0.326 | <0.001 | 20.614 | 9.872 | 177 |
| 2(C) | EXP.1 | 16℃/50 µM Ot B | 10.612±0.325 | <0.001 | 23.798 | 10.205 | 178 |
| 2(D) | EXP.2 | 16℃/Control | 9.384±0.356 |  |  | 8.673 | 159 |
|  | EXP.2 | 16℃/100µM Cuc | 10.824±0.364 | 0.008 | 15.345 | 10.539 | 153 |
|  | EXP.2 | 16℃/50 µM Ot B | 10.652±0.355 | 0.012 | 13.512 | 9.939 | 161 |
|  | EXP.3 | 16℃/Control | 8.551±0.305 |  |  | 7.729 | 158 |
|  | EXP.3 | 16℃/100µM Cuc | 10.691±0.335 | <0.001 | 25.026 | 10.061 | 165 |
|  | EXP.3 | 16℃/50 µM Ot B | 10.846±0.332 | <0.001 | 26.840 | 10.394 | 169 |
|  | EXP.4 | 16℃/Control | 8.610±0.313 |  |  | 7.818 | 154 |
|  | EXP.4 | 16℃/100µM Cuc | 10.634±0.326 | <0.001 | 23.508 | 9.883 | 164 |
|  | EXP.4 | 16℃/50 µM Ot B | 10.488±0.345 | <0.001 | 21.812 | 9.833 | 162 |

Paralysis experiments were carried out at 16℃. 100 µM Curcumin was used as a positive control. The mean paralysis time were calculated by a log-rank (Kaplan-Meier) statistical test by using SPSS package. *p* Values were calculated by two-tailed t-test. PT50 means 50% worm paralyzed. N: total number of worms in each individual experiment.

**Table S4. The effects of Ot B on paralysis of CL4176**

| **Figure** | **Strain** | **Treatment** | **24h** | **30h** | **36h** | **N** |
| --- | --- | --- | --- | --- | --- | --- |
|  | **CL4176** | | | | | |
| 3(B) | EXP.1 | Control | 0.038 | 0.800 | 0.971 | 68 |
|  | EXP.1 | 100 µM Cuc | 0.048 | 0.508 | 0.750 | 80 |
|  | EXP.1 | 50 µM Ot B | 0.054 | 0.489 | 0.735 | 68 |
|  | EXP.2 | Control | 0.053 | 0.732 | 0.918 | 61 |
|  | EXP.2 | 100 µM Cuc | 0.037 | 0.575 | 0.833 | 48 |
|  | EXP.2 | 50 µM Ot B | 0.048 | 0.580 | 0.786 | 56 |
|  | EXP.3 | Control | 0.035 | 0.667 | 0.873 | 63 |
|  | EXP.3 | 100 µM Cuc | 0.031 | 0.444 | 0.800 | 60 |
|  | EXP.3 | 50 µM Ot B | 0.052 | 0.463 | 0.840 | 50 |
|  | **Mean Lifespan** | **Control** | 0.042 | 0.733 | 0.921 |  |
|  | **SEM** |  | 0.005 | 0.038 | 0.028 |  |
|  | **Mean Lifespan** | **100 µM Cuc** | 0.039 | 0.509 | 0.794 |  |
|  | **SEM** |  | 0.005 | 0.038 | 0.024 |  |
|  | **Mean Lifespan** | **50 µM Ot B** | 0.051 | 0.511 | 0.787 |  |
|  | **SEM** |  | 0.002 | 0.035 | 0.030 |  |
|  | **P (Cur VS Control)** | | 0.702 | 0.014 | 0.028 |  |
|  | **P (Ot B VS Control)** | | 0.216 | 0.013 | 0.032 |  |

Worms were grown at 16℃ for 48h, then transferred to 25℃. Paralysis experiments were carried out at 25℃ and calculated the paralysis of worms in 24h, 30h and 36h. 100 µM Curcumin was used as a positive control. *p* Values were calculated by two-tailed t-test. N: total number of worms in each individual experiment.

**Table S5.** **The effects of Ot B on chemotaxis index of CL2122 and CL2355**

| **Figure** | **Strain** | **Treatment** | **Chemotaxis index** | **N** | **Strain** | **Treatment** | **Chemotaxis index** | **N** |
| --- | --- | --- | --- | --- | --- | --- | --- | --- |
|  | **CL2122** | | | | **CL2355** | | | |
| 3(C) | EXP.1 | Control | 0.192 | 52 | EXP.1 | Control | 0.167 | 36 |
|  | EXP.1 | 100µM Cuc | 0.256 | 43 | EXP.1 | 100µM Cuc | 0.217 | 46 |
|  | EXP.1 | 50 µM Ot B | 0.200 | 45 | EXP.1 | 50 µM Ot B | 0.211 | 38 |
|  | EXP.2 | Control | 0.234 | 47 | EXP.2 | Control | 0.190 | 42 |
|  | EXP.2 | 100µM Cuc | 0.200 | 40 | EXP.2 | 100µM Cuc | 0.237 | 38 |
|  | EXP.2 | 50 µM Ot B | 0.275 | 40 | EXP.2 | 50 µM Ot B | 0.222 | 36 |
|  | EXP.3 | Control | 0.333 | 39 | EXP.3 | Control | 0.163 | 43 |
|  | EXP.3 | 100µM Cuc | 0.289 | 38 | EXP.3 | 100µM Cuc | 0.250 | 40 |
|  | EXP.3 | 50 µM Ot B | 0.237 | 38 | EXP.4 | 50 µM Ot B | 0.214 | 42 |
|  | EXP.4 | Control | 0.229 | 35 | EXP.4 | Control | 0.182 | 33 |
|  | EXP.4 | 100µM Cuc | 0.289 | 38 | EXP.4 | 100µM Cuc | 0.265 | 34 |
|  | EXP.4 | 50 µM Ot B | 0.225 | 40 | EXP.4 | 50 µM Ot B | 0.278 | 36 |
|  | **Mean** | **Control** | 0.247 |  | **Mean** | **Control** | 0.175 |  |
|  | **SEM** |  | 0.030 |  | **SEM** |  | 0.006 |  |
|  | **Mean** | **100µM Cuc** | 0.259 |  | **Mean** | **100µM Cuc** | 0.242 |  |
|  | **SEM** |  | 0.021 |  | **SEM** |  | 0.010 |  |
|  | **Mean** | **50 µM Ot B** | 0.234 |  | **Mean** | **50 µM Ot B** | 0.231 |  |
|  | **SEM** |  | 0.016 |  | **SEM** |  | 0.016 |  |
|  | **P (Cur VS Control)** | | 0.764 |  | **P (Cur VS Control)** | | 0.002 |  |
|  | **P (Ot B VS Control)** | | 0.723 |  | **P (Ot B VS Control)** | | 0.031 |  |

Chemotaxis experiments were carried out at 23℃. 100 µM Curcumin was used as a positive control. Chemotaxis index was defined as follows: (number of worms at the attractant location – number of worms at the control location)/total number of worms on the plate. *p* values were calculated by two-tailed t-test. N: total number of worms in each individual experiment.

**Table S6. The effects of Ot B on Aβ deposition**

| **Figure** | **Strain** | **Treatment(Day 3)** | **Mean Deposits** | **N** | **Strain** | **Treatment(Day 5)** | **Mean Deposits** | **N** |
| --- | --- | --- | --- | --- | --- | --- | --- | --- |
|  | **CL2006** | | |  | **CL2006** | | |  |
| 4(B) | EXP.1 | 16℃/Control | 9.300 | 20 | EXP.1 | 16℃/Control | 13.800 | 25 |
|  | EXP.1 | 16℃/50 µM Ot B | 5.800 | 20 | EXP.1 | 16℃/50 µM Ot B | 7.826 | 23 |
|  | EXP.2 | 16℃/Control | 9.120 | 25 | EXP.2 | 16℃/Control | 13.905 | 21 |
|  | EXP.2 | 16℃/50 µM Ot B | 6.391 | 23 | EXP.2 | 16℃/50 µM Ot B | 11.700 | 20 |
|  | EXP.3 | 16℃/Control | 8.833 | 24 | EXP.3 | 16℃/Control | 14.034 | 29 |
|  | EXP.3 | 16℃/50 µM Ot B | 6.741 | 27 | EXP.3 | 16℃/50 µM Ot B | 11.050 | 20 |
| **Mean Deposits** | | **Control** | 9.083 |  |  |  | 13.913 |  |
| **SEM** | |  | 0.137 |  |  |  | 0.068 |  |
| **Mean Deposits** | | **50 µM Ot B** | 6.310 |  |  |  | 10.192 |  |
| **SEM** | |  | 0.274 |  |  |  | 1.198 |  |
| **P value VS Control** | | | 0.001 |  |  |  | 0.045 |  |

Aβ were stained with thioflavin S. Fluorescence images were acquired using a 100×objective of a fluorescence microscope. The thioflavin S-reactive deposits anterior of the pharyngeal bulb in individual animals were scored. *p* Values were calculated by two-tailed t-test, each consisting of control and experimental animals as the same time. N: total number of worms in each individual experiment.

**Table S7. The effects of Ot B on mRNA expression**

| **Figure** | **Strain** | ***Aβ*** | ***daf-16*** | ***sod-3*** | ***dod-3*** | ***sip-1*** | ***skn-1*** | ***gst-4*** |
| --- | --- | --- | --- | --- | --- | --- | --- | --- |
|  | **CL2006** | | | | | | |  |
| 4(C) | EXP.1 | 0.674 | 0.808 | 1.816 | 1.112 | 1.192 | 1.218 | 1.174 |
| 5(A) | EXP.2 | 0.771 | 0.762 | 1.714 | 0.974 | 0.842 | 0.990 | 1.093 |
| 5(B) | EXP.3 | 0.849 | 0.906 | 1.642 | 1.151 | 1.298 | 1.197 | 1.395 |
| **Mean** |  | 0.765 | 0.826 | 1.724 | 1.079 | 1.111 | 1.135 | 1.221 |
| **SEM** |  | 0.051 | 0.042 | 0.050 | 0.054 | 0.138 | 0.073 | 0.090 |
| **P value VS Control** | | 0.043 | 0.054 | 0.005 | 0.279 | 0.506 | 0.205 | 0.134 |
| **Figure** | **Strain** | ***gcs-1*** | ***nit-1*** | ***hsf-1*** | ***hsp-12.6*** | ***hsp-16.2*** | ***hsp-70*** |  |
|  | **CL2006** | | | | | | |  |
| 5(B) | EXP.1 | 1.584 | 1.499 | 1.595 | 1.779 | 2.178 | 1.523 |  |
| 5(C) | EXP.2 | 1.155 | 1.175 | 1.691 | 1.725 | 1.595 | 1.345 |  |
|  | EXP.3 | 1.266 | 1.047 | 1.709 | 1.552 | 1.838 | 1.768 |  |
| **Mean** |  | 1.335 | 1.240 | 1.665 | 1.685 | 1.870 | 1.545 |  |
| **SEM** |  | 0.129 | 0.135 | 0.035 | 0.068 | 0.169 | 0.123 |  |
| **P value VS Control** | | 0.121 | 0.216 | 0.003 | 0.010 | 0.036 | 0.047 |  |

Every value of number representatives the expression level of genes and was carried out using 2^–△△CT^ method and normalized to the expression of gene *cdc-42*. In each experiment, control and experimental worms were conducted in parallel and repeated in three independent trials. *p* Values were calculated by two-tailed t-test.

**Table S8. Primer sequences of genes used in experiment**

| **Gene** | **Type** | **Sequence** |
| --- | --- | --- |
| ***cdc-42*** | F | 5’-CTGCTGGACAGGAAGATTACG-3’ |
|  | R | 5’-CTCGGACATTCTCGAATGAAG-3’ |
|  |  |  |
| ***Aβ*** | F | 5’-CCGACATGACTCAGGATATGAAGT-3’ |
|  | R | 5’-CACCATGAGTCCAATGATTGCA-3’ |
| ***daf-16*** | F | 5’- CCAGACGGAAGGCTTAAAACT -3’ |
|  | R | 5’- ATTCGCATGAAACGAGAATG-3’ |
| ***sod-3*** | F | 5’-AGCATCATGCCACCTACGTGA-3’ |
|  | R | 5’-CACCACCATTGAATTTCAGCG-3’ |
| ***dod-3*** | F | 5’-AAGCCATGTTCCCGAATGAG-3’ |
|  | R | 5’-GCTGCGAAAAGCAAGAAAATG-3’ |
| ***sip-1*** | F | 5’-TTCGAGGACATGATGCCATA-3’ |
|  | R | 5’-TGAACGATCTCTTGCTGAACC-3’ |
| ***skn-1*** | F | 5’-AGTGTCGGCGTTCCAGATTTC-3’ |
|  | R | 5’-GTCGACGAATCTTGCGAATCA-3’ |
| ***gst-4*** | F | 5’-TCCGTCAATTCACTTCTTCCG-3’ |
|  | R | 5’-AAGAAATCATCACGGGCTGG-3’ |
| ***gcs-1*** | F | 5’-CCCATTTTACAAGTCGATGG-3’ |
|  | R | 5’-CTTCCTCTGCAGTTTTTCCA-3’ |
| ***nit-1*** | F | 5’-AATCCTCCGACTATCCCTTG-3’ |
|  | R | 5’-AGCGAATCGTTTCTTTTGTG-3’ |
| ***hsf-1*** | F | 5’-TTGACGACGACAAGCTTCCAGT-3’ |
|  | R | 5’-AAAGCTTGCACCAGAATCATCCC-3’ |
| ***hsp-12.6*** | F | 5’-GTGATGGCTGACGAAGGAAC-3’ |
|  | R | 5’-GGGAGGAAGTTATGGGCTTC-3’ |
| ***hsp-16.2*** | F | 5’-CTGCAGAATCTCTCCATCTGAGTC-3’ |
|  | R | 5’-AGATTCGAAGCAACTGCACC-3’ |
| ***hsp-70*** | F | 5’-CGTTTCGAAGAACTGTGTGCTGATCTATTCCGG-3’ |
|  | R | 5’-TTAATCAACTTCCTCAACAGTAGGTCCTTGTGG-3’ |
